# Disability and quality of life in patients with different forms of migraine

**DOI:** 10.1186/1129-2377-16-S1-A4

**Published:** 2015-09-28

**Authors:** Domenico D'Amico, Matilde Leonardi, Licia Grazzi, Marcella Curone, Alberto Raggi

**Affiliations:** Headache and Neuroalgology Unit, Neurological Institute C. Besta, IRCCS Foundation, Milan, Italy; Neurology, Public Health and Disability Unit, Neurological Institute C. Besta, IRCCS Foundation, Milan, Italy

## Background and state of the art

Migraine (M) is the seventh leading cause of years of life lost to disability (YLDs) worldwide, responsible for 2.9% of all YLDs, more than half of all YLDs attributable to neurological disorders[1]. Its negative effects result from several studies, particularly in surveys carried out by our research group, through the application of patient oriented outcome measures (PROMS), and in their development and validation[2–4]. Administration of MIDAS (a migraine-specific tool) demonstrated that: the disability level is rather high (particularly in chronic M); social, family and leisure activities are more impaired than work activities; days spent at work with reduced effectiveness are more than days of absence[3–5]. A better understanding of the pervasive impact of migraine has been achieved using the WHODAS 2.0, a questionnaire based on the International Classification of Functioning, which captures the interaction between the individual's health status and the context of life. Our data, together with many results deriving from the application of quality of life tools (e.g., SF-36 and MSQ), showed that migraine influences physical and emotional domains, causes restriction and avoidance of activities also outside the headache episodes[6–11].

## Measuring the impact of migraine: a relevant issue in different fields

Migraine has to be considered both as a clinical and as a public health problem: therefore, at least two perspectives need to be taken into account:

1. Epidemiological and healthcare perspectives: to evaluate the burden of migraine in the population, understand patients’ needs in implementing appropriate treatments and address healthcare interventions.

2. Clinical practice and research perspectives: to assess the severity of migraine in an individual patient, to better tailor treatment plans, and eventually to evaluate their global outcome, and to assess the efficacy of new treatments in RCTs and observational studies.

## Concluding remarks and future directions

Migraine is a common condition, whose impact is relevant on individuals, in terms of personal suffering and reduced health, and on societies, in terms of reduced productivity and increased costs for the health system[7, 8, 12]. The systematic use of PROMs should be encouraged both in the clinical and in the research fields, particularly in RCTs to quantify the potential benefits of treatments. Further (ongoing) research is needed to understand the potential application of the different tools in the different contexts, as well as to develop new instruments to assess the qualitative aspects of migraine-related disability, particularly in the workplace.

## Conflict of interest

None declared.
